# Supplementary material for: Engineered Sleeping Beauty Transposon as Efficient System to Optimize Chimp Adenoviral Production
Source: Int J Mol Sci. 2022 Jul 7;23(14):7538. doi: 10.3390/ijms23147538 (PMC9316264; doi:10.3390/ijms23147538)
Supplement: Supplementary file 1 [file ijms-23-07538-s001.zip › ijms-1652589-supplementary.pdf]

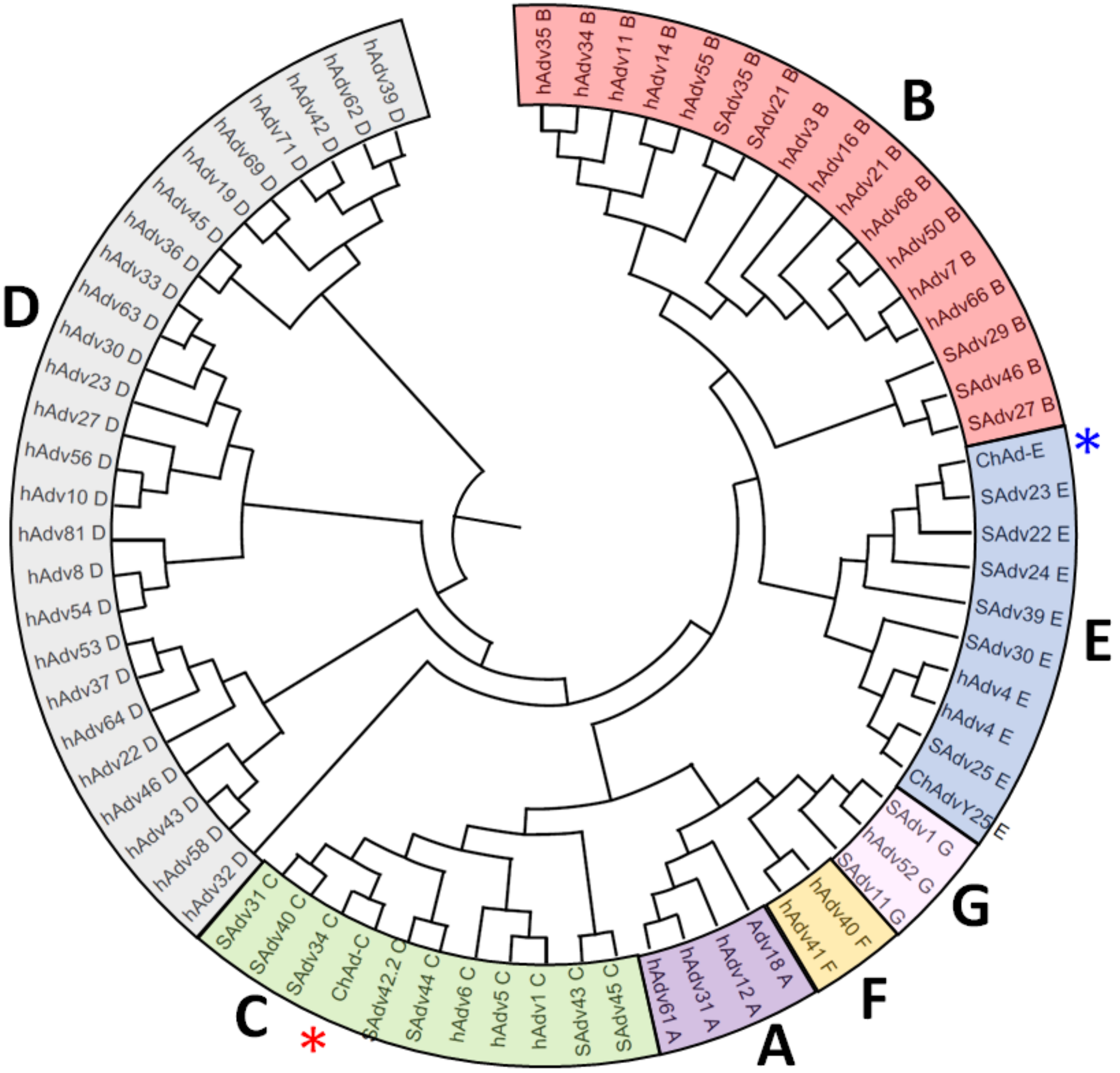

Figure S1 Phylogenetic tree. Alignment of hexon protein obtained using the Neighbor-Joining method with 1000 bootstrap replications. Human Mastadenovirus species are labelled A to G. ChAd-C and ChAd-E cluster with human Masteadenovirus species C and E, respectively.

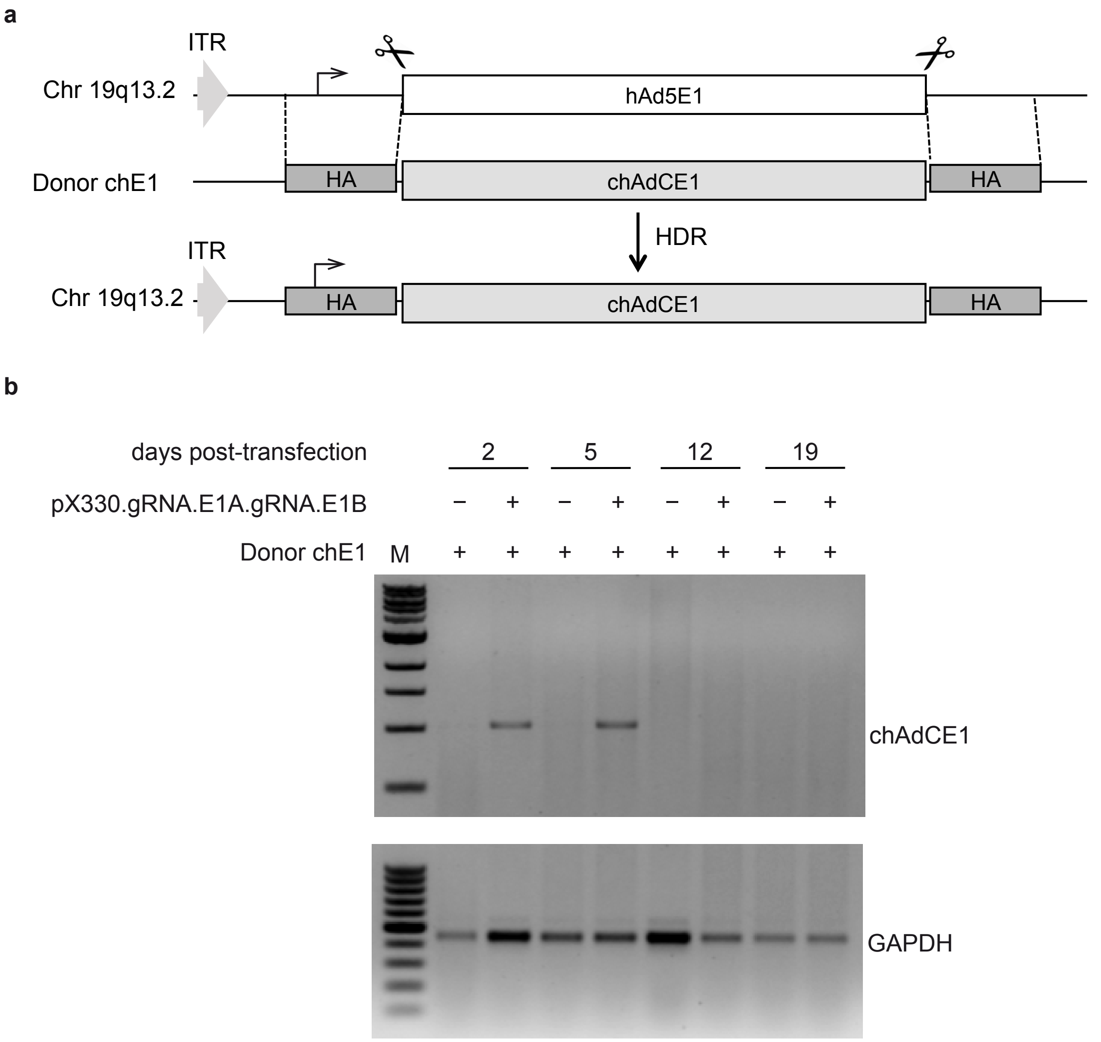

Figure S2. CRISPR-mediated replacement of hAd5 E1 with chAd-C E1. a) Top: Schematic representation (not in scale) of hAd5 5’-terminus, from inverted terminal repeat (ITR, gray arrow) to the end of hAd5E1 gene, integrated in chromosome 19 of HEK293 cells. Scissors indicate gRNA.E1A and gRNA.E1B cut sites. The black arrow indicates the transcription start site of hAd5 E1 gene. Middle: schematic representation (not in scale) of promoterless chAd-C E1 donor cloned between 5’- and 3’ homology arms (HA). Bottom: HDR event into the target site leads to the replacement of hAd5 E1 with chAd-C E1 in the genomic DNA of HEK293 cells. **b)** Targeted integration PCR analysis on genomic DNA from HEK293 cells transfected with the donor chE1 in presence or absence of pX330gRNA.E1A.E1B. The housekeeping GAPDH gene was amplified as control. M, 1 kb molecular weight marker.

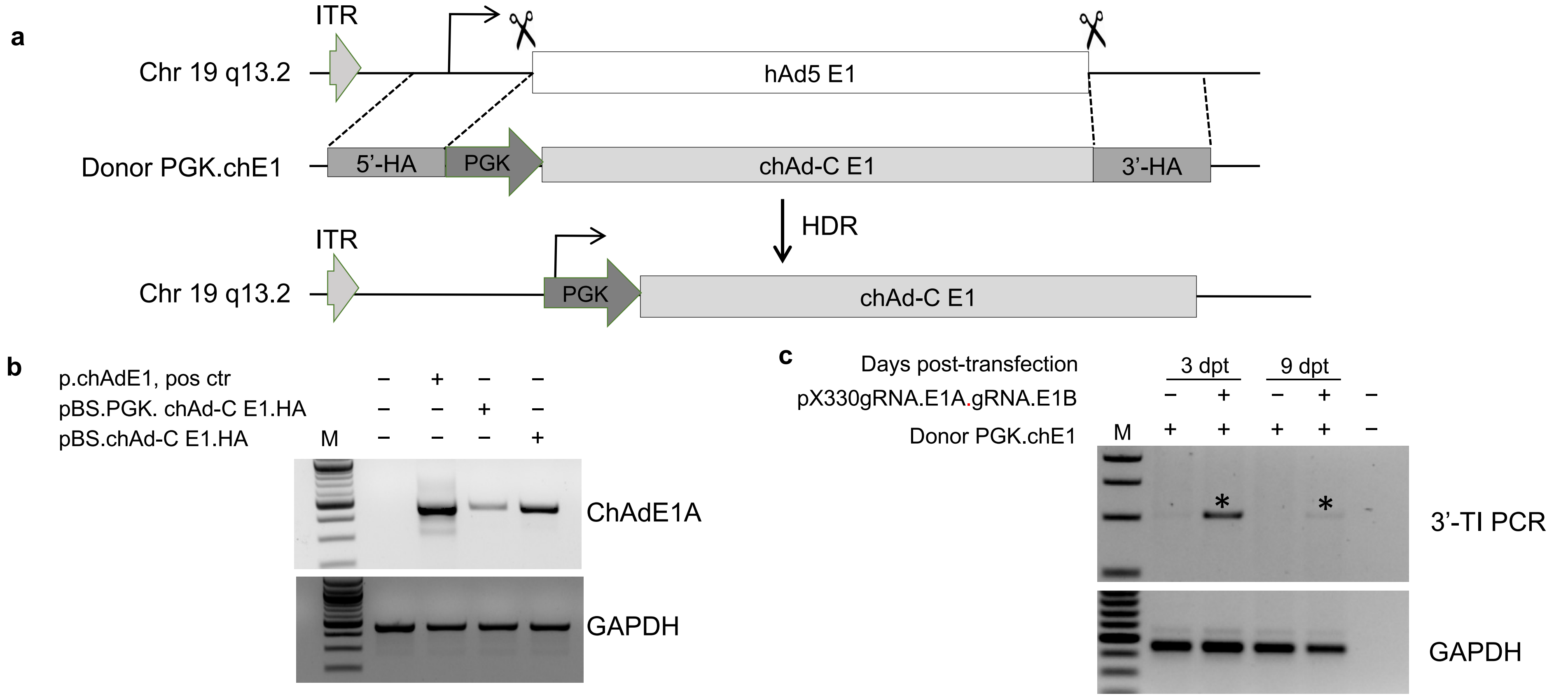

Figure S3. CRISPR-mediated replacement of hAd5 E1 with PGK.chAd-C E1 expression cassette in HEK293 cells. a) Top: Schematic representation (not in scale) of 5'-term of hAd5 genomic region integrated in chromosome 19 of HEK293 cells, from inverted terminal repeat (ITR) to hAd5 E1. Scissors indicate gRNA.E1A and gRNA.E1B cut sites. The arrow indicates the transcription star site of hAd5 E1. Middle: schematic representation of PGK.ChAd-C E1 donor cloned between 5'- and 3' homology arms (HA). Bottom: HDR events into the target site lead to the replacement of hAd5E1 with PGK.chAd-C E1 expression cassette in the genomic DNA of HEK293 cells. **b)** Analysis of transcript expression of chAdE1 in HDR donors. Semi-quantitative RT-PCR analysis of chAdE1 in HeLa cells transfected with pBS.PGK.chAd-C E1.HA or pBS.chAd-C E1.HA or a positive control vector (p.chAdE1, pos ctr) carrying the promoter/regulatory and coding sequences of the chAdE1. The expression of *GAPDH* was used for normalization. M, molecular weight marker. **c**) PCR analysis on genomic DNA from HEK293 cells co-transfected with pBS.PGK.chAd-C E1.HA and pX330gRNA.E1A-2.E1B or not, extracted 3 and 9 days post-transfection (dpt). A couple of primers specific for 3' donor-genome junction amplified a 1029-bp band indicated by a star.

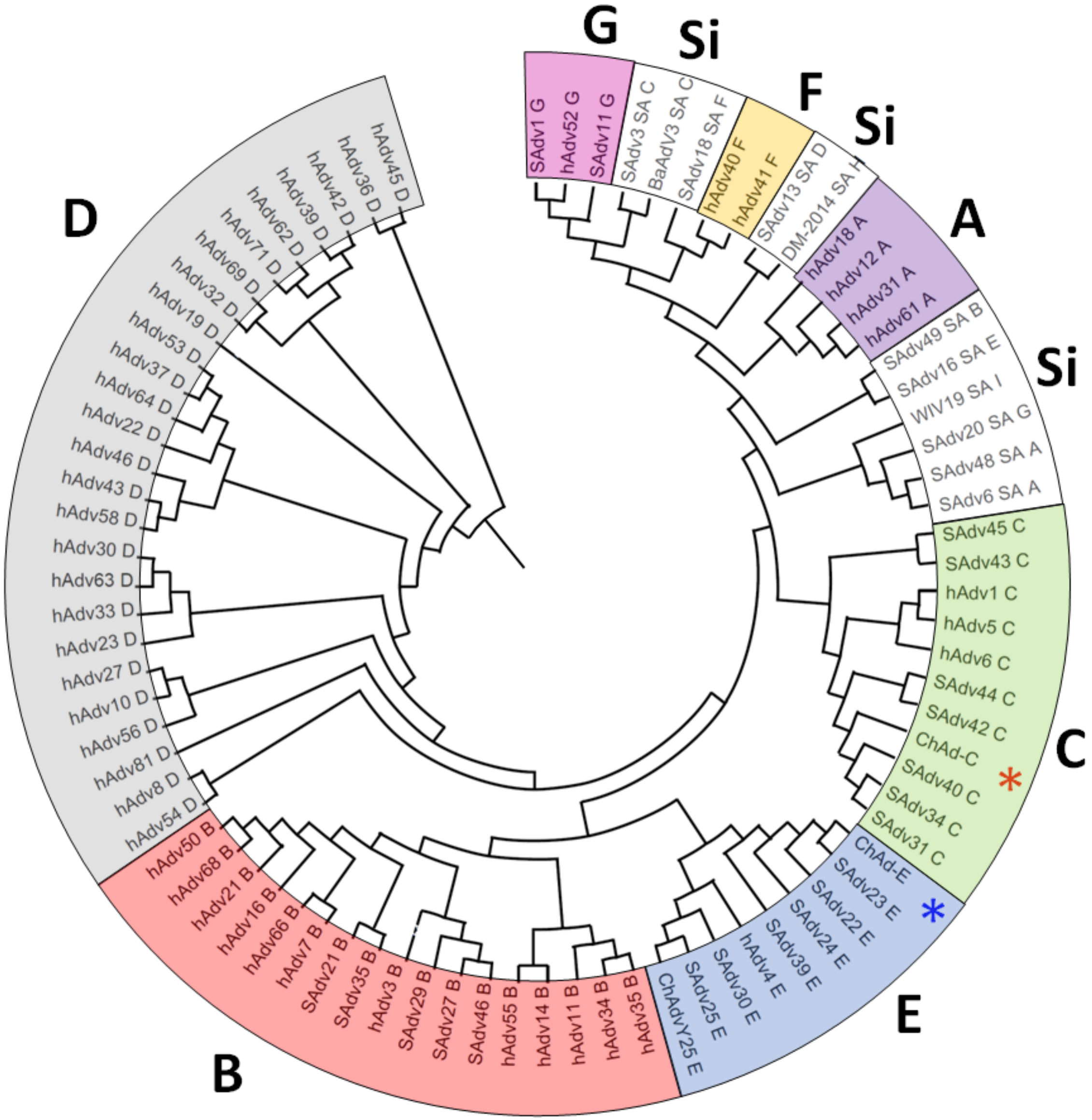

Figure S4 Phylogenetic tree. Alignment of pTP proteins obtained using the Neighbor-Joining method with 1000 bootstrap replications. Human Mastadenovirus species are labelled A to G, Simian Mastadenovirus species are indicated by Si. ChAd-C and ChAd-E cluster with human Masteadenovirus species C and E, respectively.

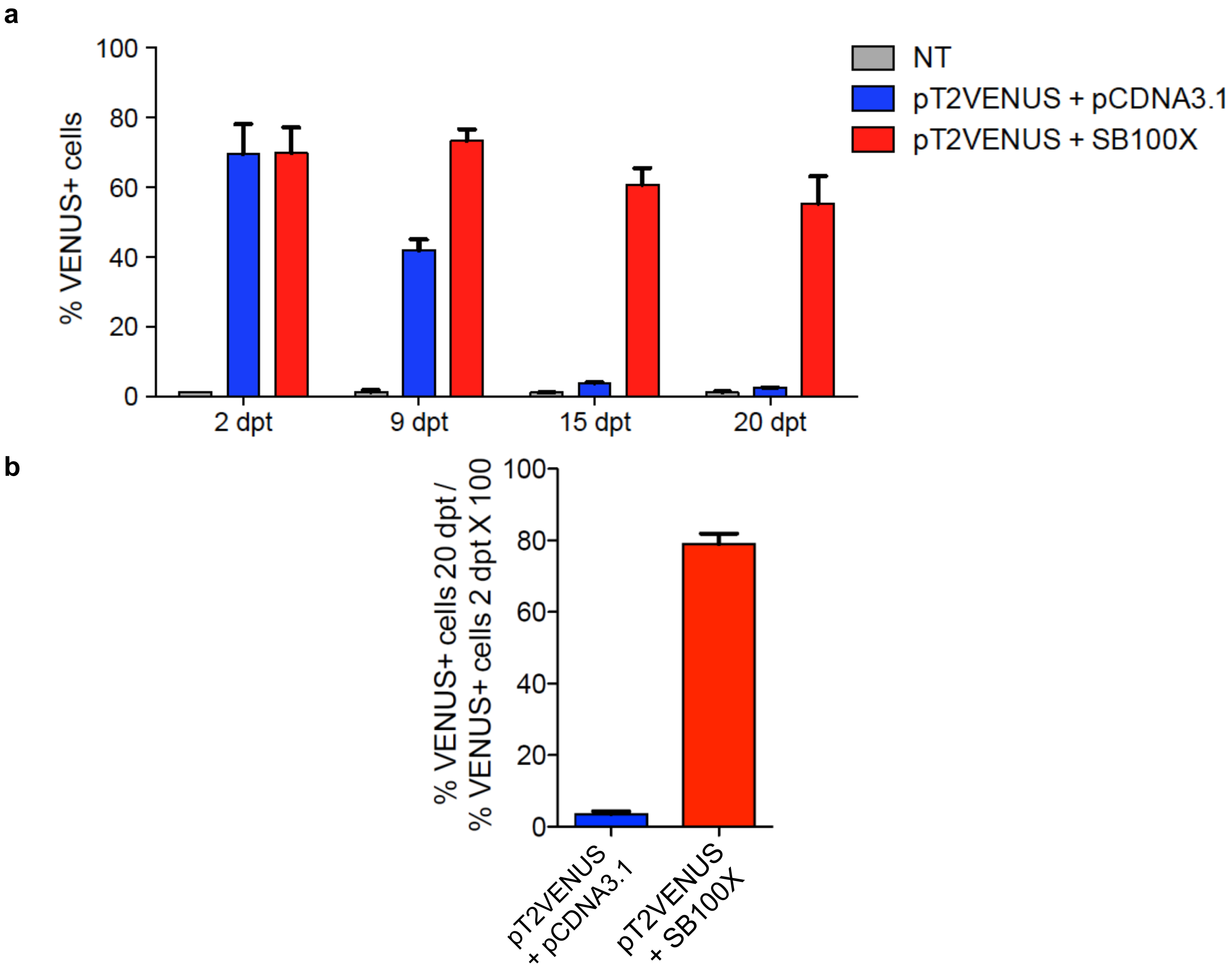

Figure S5. Transposition mediated by Sleeping Beauty (SB) transposases in HEK293 cells. a) Flow cytometric analysis of HEK 293 cells transfected with pT2VENUS transposon plasmid together with pCMVSB100X (SB100X) transposase plasmid or control (pCDNA3.1). The expression of VENUS^{+} cells was analyzed 2, 9, 15, 20 days post-transfection (dpt). NT, not treated cells. The experiment was performed in duplicate. **b)** Transposition efficiency in HEK293 cells transfected with pT2VENUS transposon plasmid together with pCMVSB100X (SB100X) transposase plasmid or control (pCDNA3.1), presented as %VENUS^{+} cells 20 dpt / %VENUS+ cells 20 dpt X 100 (mean ± SEM).

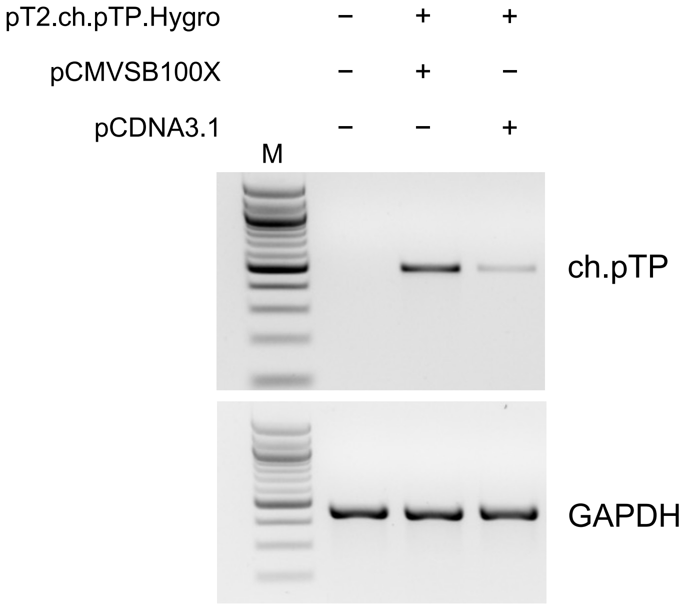

Figure S6. Semi-quantitative analysis of ch.pTP expression in Hygro^{R} HEK293 cells. RT-PCR analysis to asses the expression of ch.pTP in HEK293 cells co-transfected with pT2.ch.pTP.Hygro and pCMVSB100X or pCDNA3.1. The analysis was performed 26 days post-transfection. The expression of GAPDH was used for normalization. M, 100 bp molecular weight marker.

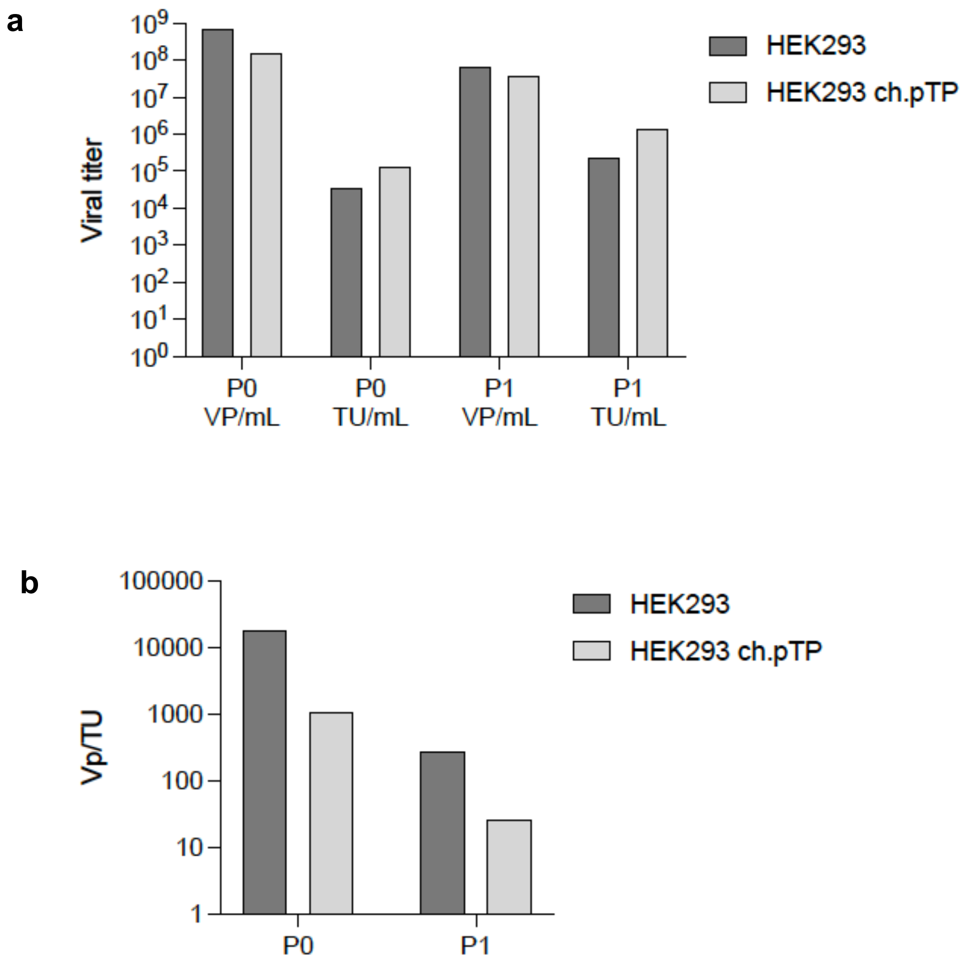

Figure S7. ChAd-E EGFP rescue and replication. a) Rescue (P0) and amplification (P1) of chAd-E EGFP vector in HEK293 and HEK293ch-pTP cells. Titration of VP/ml and TU/ml is indicated. **b**) Ratio VP/TU as a parameter of the quality of chAd-E vector rescued (P0) and amplified (P1) in HEK293 and HEK293 ch.pTP cells.

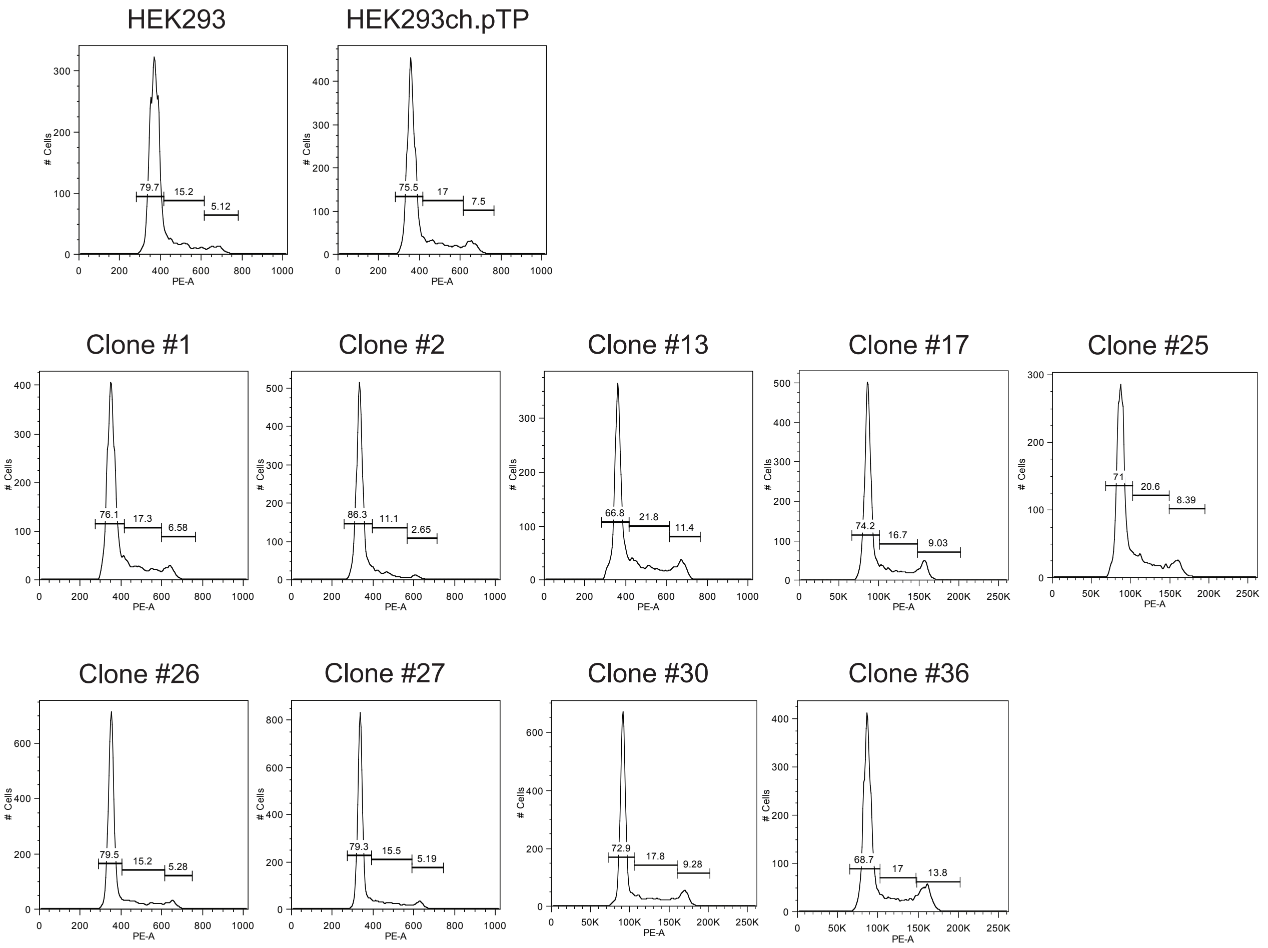

Figure S8. Cell cycle analysis of clones isolated from HEK293 ch.pTP bulk. Cell cycle analysis was performed by propidium iodide staining of nine clones (#1, #2, #13, #17, #25, #26, #27, #30, #36) clones, HEK293 cells and HEK293ch-pTP bulk cultured for 7 days.

Table S1. Rescue and replication of chAd-C vector in HEK293ch.pTP bulk frozen at different time points after pTP transposition

		chAd-C Experiment I		chAd-C Experiment II	
**Passage**	**Packaging cells**	**VP/mL**	**TU/mL**	**VP/mL**	**TU/mL**
P0	HEK293	4.53E+08	4.83E+05	6.75E+08	2.27E+06
	HEK293ch.pTP 19d	2.83E+09	4.04E+07	6.35E+09	7.17E+07
	HEK293ch.pTP 30d	5.53E+09	4.64E+07	5.15E+09	5.03E+07
	HEK293ch.pTP 48d	8.75E+09	4.84E+07	5.10E+09	4.17E+07
P1	HEK293	4.44E+08	5.29E+05	9.33E+08	2.88E+07
	HEK293ch.pTP 19d	1.83E+09	1.99E+08	3.44E+09	6.21E+08
	HEK293ch.pTP 30d	6.68E+09	1.42E+08	3.89E+09	4.88E+08
	HEK293ch.pTP 48d	1.62E+09	6.55E+07	3.07E+09	1.24E+09

Table S2. Primers used in the study.

Primer ID	Primer sequence (5' to 3')	Application
gRNA-E1A-542-F	CACCGTCATTTTCAGTCCCGGTGT	Oligo annealing for gRNA.E1A
gRNA-E1A-542-RC	AAACACACCGGGACTGAAAATGAC	Oligo annealing for gRNA.E1A
gRNA-E1B-3510_F	CACCGGTACTGAAATGTGTGGGCG	Oligo annealing for gRNA.E1B
gRNA-E1B-3510_R	AAACCGCCCACACATTTCAGTACC	Oligo annealing for gRNA.E1B
ChAdE1-3HA-F2	GACACAGATTGAGTCGACTAAGGGTG	3-TI PCR
PSG4-RC	AGCCACATTTCCCCTGAGATGTTACG	3-TI PCR
CAG-F	GTCCCCTTCTCCATCTCCAG	PCR for integration of pTP cassette and RT-PCR
pTP-RC	CATCACGCTCCAATTCACGG	PCR for integration of pTP cassette and RT-PCR
ChAdE1-F	CTAGCTAGCTCTCCGCTCCGCTCCGC	RT-PCR
ChAdE1-R	TCACCCTCTTCATCCTCGTC	RT-PCR
GAPDH-F	GACCACAGTCCATGCCATCAC	PCR and RT-PCR
GAPDH-R	TCCACCACCCTGTTGCTGTAG	PCR and RT-PCR
CMV promoter Fw	CATCTACGTATTAGTCATCGCTATTACCA	Primer for digital droplet PCR
CMV promoter Rw	GACTTGGAAATCCCCGTGAGT	Primer for digital droplet PCR
CMV promoter Probe	ACATCAATGGGCGTGGATAGCGGTT	Probe for digital droplet PCR
ch.pTP-F	AGTCCGCCTACTTCAACTACATCA	Primer for Taqman RealTime RTqPCR
ch.pTP-R	GGCTGGAGGTGGAGGATGA	Primer for Taqman RealTime RTqPCR
ch.pTP-probe	AGCGCCAGACACAG	Probe for Taqman RealTime RTqPCR
pTP-SYBR-F	ATGGCCAACTGCACCTACAC	Primer for SYBR green RealTime RTqPCR
pTP-SYBR-R	CAGGATGGCCTGCTGCAC	Primer for SYBR green RealTime RTqPCR
SYBR-B-ACTIN-F	CAGAAGGATTCCTATGTGG	Primer for SYBR green RealTime RTqPCR
SYBR-B-ACTIN-R	CATGATCTGGGTCATCTTC	Primer for SYBR green RealTime RTqPCR
